# Percutaneous transhepatic cholangioscopy-guided holmium laser lithotripsy for bile duct stones in elderly patients

**DOI:** 10.1371/journal.pone.0356488

**Published:** 2026-08-24

**Authors:** Nhan Hien Phan, Dang Khanh Do, Hong Canh Pham, Thai Binh Nguyen

**Affiliations:** 1 Radiology Center, Hanoi Medical University Hospital, Hanoi Medical University, Hanoi, Vietnam; 2 Radiology Department, Hanoi Medical University, Hanoi, Vietnam; Biology and Health Laboratory, Ibn Tofail University, MOROCCO

## Abstract

**Purpose:**

To evaluate the safety and efficacy of percutaneous transhepatic endoscopic holmium laser lithotripsy (PTEHL) for bile duct stones in elderly patients aged ≥80 years.

**Materials and methods:**

This retrospective study included 50 patients (mean age 86.4 ± 4.5 years) who underwent PTEHL from January 2023 to December 2024. Inclusion criteria were age ≥ 80 years, presence of intra- and/or extrahepatic bile duct stones, and indication for PTEHL. Outcomes assessed included stone clearance, adverse events, and hospitalization duration. Adverse events were categorized according to the CIRSE classification.

**Results:**

PTEHL was performed once in 48 patients, while 2 patients required two sessions. Final outcomes showed that all patients achieved complete (72%) or near-complete stone clearance (28%), with no requirement for any additional subsequent treatment. Thirteen patients (26%) experienced adverse events, mostly minor; only one moderate adverse event (biliopleural fistula) was recorded. No procedure-related mortality occurred. Multivariate analysis revealed that the presence of cholangitis and combined intra- and extrahepatic stones were significantly associated with incomplete clearance. Mean hospital stay was 14.8 ± 8.0 days and was significantly longer in patients with cholangitis (p = 0.001).

**Conclusion:**

PTEHL may represent a feasible minimally invasive treatment option for elderly patients with biliary stone.

## Introduction

Cholelithiasis is common in the elderly, with prevalence rising from 14–23% in individuals aged 65 years and older to over 80% in those over 90 years. In this population, clinical symptoms may be vague, yet complications such as cholangitis, septic shock, and organ failure are more frequent and carry higher morbidity and mortality, especially in patients with comorbidities [1,2]. Age-related anatomical changes and frailty further increase perioperative risks [1–3].

Endoscopic Retrograde Cholangiopancreatography (ERCP) is the standard for common bile duct (CBD) stones but may be less effective in large or intrahepatic stones and poses higher complication risks in older adults [4,5]. Altered anatomy from previous surgery can limit ERCP access. Surgery, while effective, may not be feasible due to anesthesia risks and frailty [6–8].

Temporary drainage via ERCP or PTBD relieves obstruction but does not clear stones. Definitive stone clearance is essential to prevent recurrent infection and improve outcomes [6,9]. Holmium laser lithotripsy, well established in urology, has shown promise in biliary stone management via the percutaneous transhepatic approach, particularly in patients with existing drainage tracts [10–12]. Percutaneous transhepatic endoscopic holmium laser lithotripsy (PTEHL) enables minimally invasive treatment without general anesthesia, with high success rates and low complication risk [7,11–13]. This study evaluates the safety and efficacy of PTEHL in patients aged 80 years and older with biliary lithiasis.

## Materials and methods

### Patient selection

**Fig 1:**
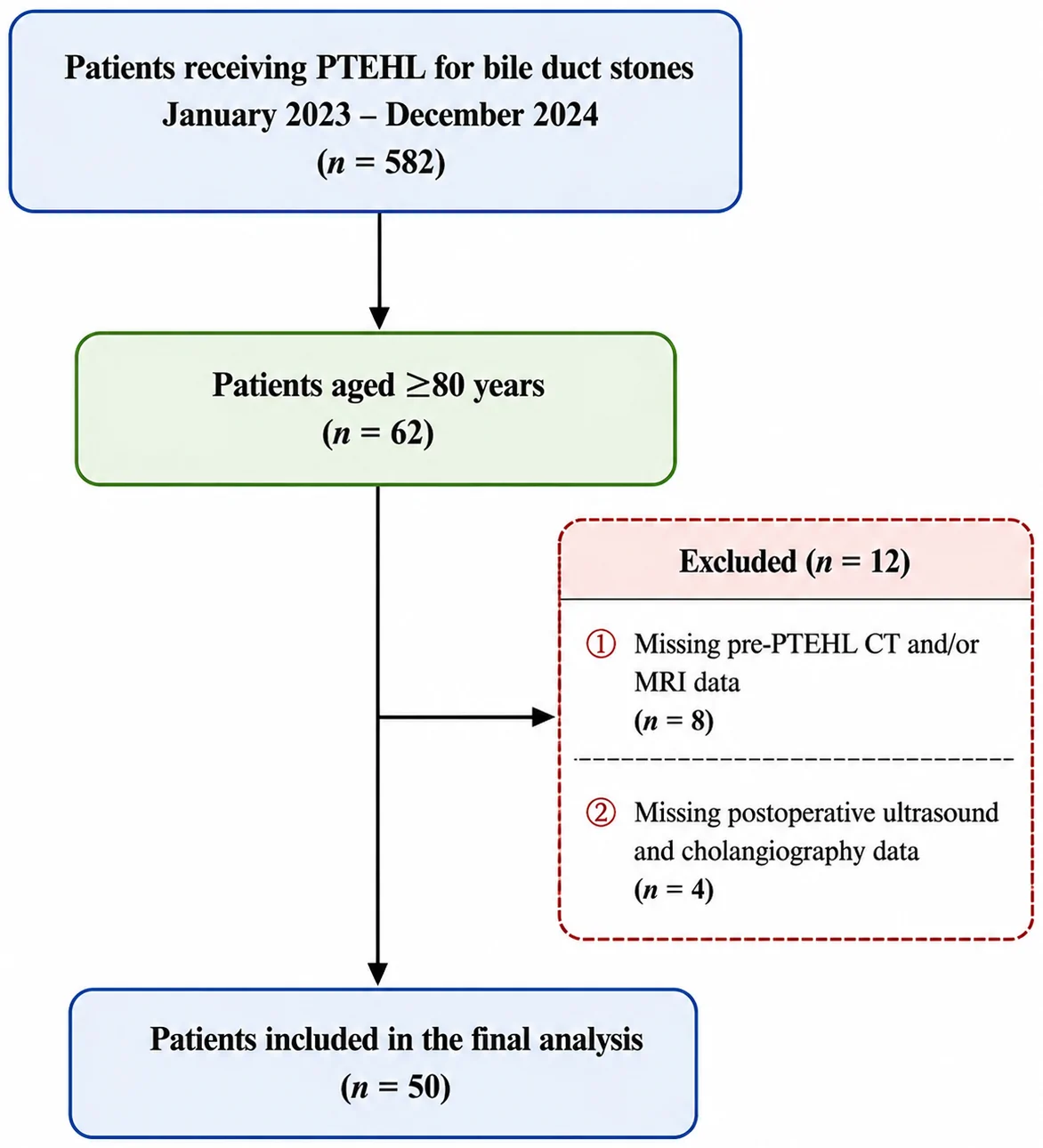
STROBE flow diagram of patient selection. Among 582 patients who underwent PTEHL for bile duct stones between January 2023 and December 2024, 62 patients were aged ≥80 years. Of these, 12 patients were excluded because of missing pre-PTEHL CT and/or MRI data *(n = 8)* or missing postoperative ultrasound and cholangiography data *(n = 4)*. Finally, 50 patients met the eligibility criteria and were included in the final analysis.

This retrospective study included patients who underwent PTEHL at our institution between January 1, 2023, and December 30, 2024. Medical records were accessed on February 1, 2025. The study protocol was approved by the Institutional Review Board of Hanoi Medical University (Approval No. HMUIRB1629). The requirement for informed consent was waived due to the retrospective nature of the study. At our institution, the selection of patients for PTEHL, ERCP, or surgical management was determined through a multidisciplinary team discussion involving specialists in internal medicine, gastrointestinal endoscopy, hepatopancreatobiliary surgery and interventional radiology. The inclusion criteria for PTEHL in patients aged ≥80 years were as follows: (1) patients with cholangitis who had previously undergone biliary drainage; (2) patients with significant systemic comorbidities precluding general anesthesia; (3) patients with complex biliary stones involving both intrahepatic and extrahepatic bile ducts; and (4) patients with common bile duct stones measuring >15 mm. Exclusion criteria comprised: (1) known malignancy of the bile ducts or gallbladder; (2) other active progressive malignancy; (3) severe coagulopathy; (4) severe systemic infection; and (5) isolated intrahepatic stones associated with significant atrophy of the corresponding hepatic parenchyma (Fig 1).

### Procedure

PTEHL was performed by interventional radiologists with over 10 years of hepatobiliary intervention experience (P.N.H, P.H.C, N.T.B). Access route was selected based on MRCP and ultrasound, prioritizing the most direct path while avoiding transpleural trajectories. The right or common hepatic duct was used for isolated CBD stones, whereas a contralateral approach was preferred for intrahepatic stones. In complex cases, multiple tracts were established.

Lithotripsy timing depended on cholangitis severity. In Tokyo grade II-III cholangitis cases, initial biliary drainage was performed, with lithotripsy delayed until infection control. In mild or non-cholangitis cases, single-session lithotripsy was considered after interdisciplinary risk assessment.

Biliary drainage was done under local anesthesia; lithotripsy utilized local anesthesia with IV sedation and monitored anesthesia care. After infiltration with 2% Lidocaine, biliary access was achieved using an 18G needle (BD Angiocath). Contrast injection confirmed ductal anatomy and stone burden. A 0.035-inch guidewire was advanced, and an 8.5 F drainage catheter (Merit) was positioned into the CBD. Bile samples were collected for culture and sensitivity testing.

**Fig 2:**
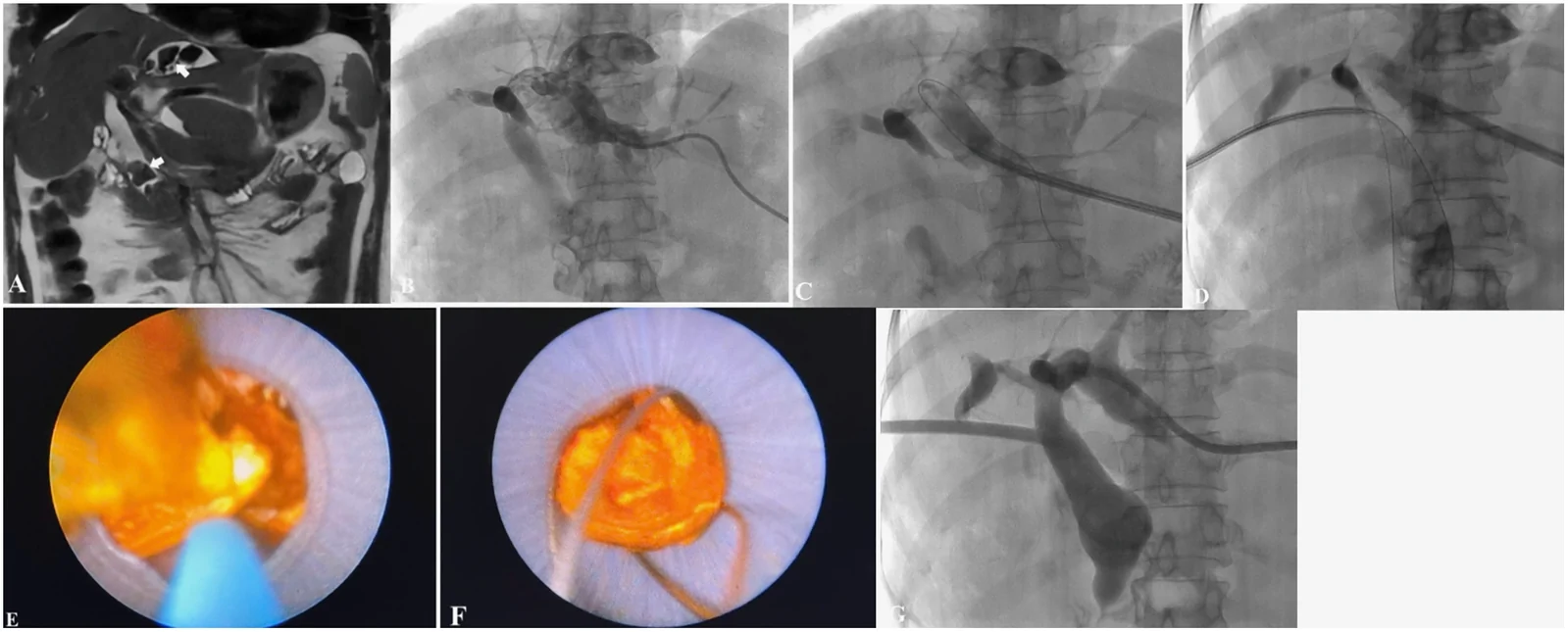
An 87-year-old woman presented with a 3-day history of abdominal pain, fever, and jaundice, diagnosed as Grade III acute cholangitis per Tokyo Guidelines. (A) Coronal T2-weighted MRI revealed multiple stones in the left hepatic duct and common bile duct (arrowhead). (B) PTBD was performed for decompression and bile culture. (C) After 7 days, percutaneous transhepatic endoscopic holmium laser lithotripsy (PTEHL) was initiated; the tract was dilated to 16F and an Amplatz sheath inserted. (D) A second tract was established into the common hepatic duct. (E-F) Holmium laser fragmented large stones, which were extracted with a retrieval basket. (G) Final cholangiography showed complete stone clearance.

For lithotripsy, tract dilation was performed using 8–16 F dilators, followed by placement of a 16–18 F Amplatz sheath (Seplou Medical). A 9.5 F rigid endoscope (Karl Storz) was introduced, and Holmium laser (80–100 W, 2 J, 20 Hz) (Accutech) was used for stone fragmentation. Fragments were retrieved with baskets and flushed via continuous irrigation. Endoscopic inspection was done from intrahepatic ducts to CBD, with biopsy of suspicious lesions. A completion cholangiogram was performed before placement of a new internal-external biliary catheter (Fig 2).

In elderly patients, multistage lithotripsy was employed to shorten procedure time and minimize fluid-related complications. Post-procedure, patients were monitored for signs of bleeding, bile leak, or fluid collection. Ultrasound was performed routinely at 24 hours or upon symptoms. Prior to discharge, repeat cholangiography and ultrasound were used to confirm clearance. The drainage catheter remained for 2–3 weeks and was removed only after imaging confirmed no residual stones.

For strictures permitting guidewire passage, options included tract dilation, balloon, incision, or large-caliber catheter placement. In high-grade strictures without wire passage, definitive treatment was deferred. Antibiotic regimens were based on bile culture when available; otherwise, broad-spectrum IV antibiotics were administered for five days post-intervention, with pre-procedural prophylaxis in non-cholangitis cases.

### Study parameters

Clinical symptoms, comorbidities (particularly chronic systemic diseases), and laboratory parameters were collected. Stone number, size, and location were evaluated via pre-procedural MRCP. Cholangitis severity was graded using the Tokyo classification (grade I – III) based on clinical and lab findings, which also informed treatment planning [14]. Data on biliary drainage duration and bile culture results were recorded.

Intra-procedural variables included number of access tracts, lithotripsy sessions, and complications. Complications such as bleeding, bile leak, or pleural effusion were classified by severity per the CIRSE classification, based on the need for intervention or observation [15]. Lithotripsy outcomes were assessed by endoscopic and cholangiography evaluation before discharge and prior to catheter removal.

Stone clearance was categorized as complete when no residual stones were detected in any bile duct on final cholangiography and cholangioscopy, near-complete clearance was defined as residual stones measuring <5 mm confined to peripheral bile duct branches distal to the subsegmental ducts, with no residual stones in the subsegmental, segmental, lobar, or main bile ducts, and no requirement for further stone-removal treatment. Technical success was defined as complete or near-complete clearance without the need for additional intervention or surgery. Treatment failure was defined as persistent obstructive or common bile duct stones requiring alternative management, including long-term drainage, stenting, or surgery. Hospital stay was calculated from initial biliary drainage (two-stage) or lithotripsy (single-stage) to discharge.

### Statistical analysis

Quantitative data with normal distribution were expressed as mean ± standard deviation (SD), and non-normally distributed data were expressed as median and interquartile range (IQR). Logistic regression analysis was used for binary outcomes, reporting odds ratios (OR) and 95% confidence intervals (CI). A p-value < 0.05 was considered statistically significant. Statistical analysis was performed using IBM SPSS Statistics version 22.0.

## Results

From January 2023 to December 2024, a total of 50 patients aged 80 years or older underwent PTEHL. The mean age of the study population was 86.4 ± 4.5 years (range: 80–100 years), with 35 female patients (70%). The most common clinical symptom was abdominal pain, reported in 48 patients (98%), followed by fever in 23 patients (46%) and jaundice in 14 patients (28%). According to the Tokyo classification, 30 patients (60%) were admitted with cholangitis, of whom 4 patients (8%) had severe cholangitis (grade III) and 21 patients (42%) had moderate cholangitis (grade II), and 5 patients (10%) with mild cholangitis (grade I). Among these, 24 patients (4 with grade III and 20 with grade II) received biliary drainage before PTEHL, with a mean drainage duration of 10.5 days.

The average stone size was 21 ± 12.7 mm (7–64 mm), with 16 patients (32%) having stones larger than 2 cm in diameter. In terms of stone count, 32 patients (64%) had multiple stones, while 18 patients (36%) had a single stone. Regarding stone location, 24 patients (48%) had isolated common bile duct stones, 23 patients (46%) had both CBD and IHBD stones, and 3 patients (6%) had only IHBD stones: one in the right hepatic duct, one in the left, and one bilaterally, (Table 1).

**Table 1:** Baseline characteristics of the study population.

Characteristics	Number (%) or mean ± SD
Age (mean ± SD)	86.4 ± 4.5
Gender (Male/Female)	15/35(30/70)
Comorbidity	
Cardiovascular disease	10 (25)
Respiratory disease	4 (8)
Renal disease	2 (4)
Clinical symptoms	
Abdominal pain	48 (96)
Fever	23 (46)
Jaundice	14 (28)
Laboratory Findings	
WBC (×10^9^/L)	11.8 ± 6.7
PLT (×10^9^/L)	230.7 ± 86.5
PT%	88.2 ± 16.5
INR	1.10 ± 0.28
Total bilirubin (µmol/L)	43.3 ± 45.4
Direct bilirubin (µmol/L)	29.5 ± 33.9
AST (U/L)	116.3 ± 18.4
ALT (U/L)	186.6 ± 39.5
eGFR mL/min/1.73 m^2^)	73.8 ± 17.1
Glucose(mmol/L)	7.53 ± 4.09
Cholangitis	
Yes	30 (60%)
No	20 (40%)
Tokyo classification	
Grade I	5 (10)
Grade II	21 (42)
Grade III	4 (8)
Stone location	
Isolated CBD	24 (48)
IBBD and CBD	23 (46)
Isolated IBBD	3 (6)
Stone size	
Mean stone size (mm)	21 ± 12.7
Stone size < 2 cm	34 (68)
Stone size ≥ 2 cm	16 (32)
Number of stones	
Single	32 (64)
Multiple	18 (36)
Biliary Stricture	
Yes	16 (32)
No	34 (68)

CBD, Common bile duct; IHBD, intrahepatic bile duct; WBC, white blood cell count; AST, aspartate aminotransferase; ALT, alanine aminotransferase; Tokyo Classification refers to diagnostic criteria for severity of acute cholangitis; PLT, Platelet count; PT, Prothrombin activity; INR, International normalized ratio; eGRF, Estimated glomerular filtration rate.

PTEHL was performed once in 48 patients, while 2 patients required two sessions. Final outcomes showed that all patients achieved complete or near-complete stone clearance, with no requirement for any additional subsequent treatment. Complete stone clearance was achieved in 36 patients (72%), while 14 patients (28%) near-complete stone clearance (Table 2). Univariate analysis identified significant factors associated with complete clearance: presence of cholangitis, biliary stricture, combined intra- and extrahepatic stones, and stone size ≥ 2 cm. In multivariate analysis, cholangitis (p = 0.05) and presence of both intra- and extrahepatic stones (p = 0.034) remained statistically significant predictors, (Table 3).

**Table 2:** Characteristics of percutaneous transhepatic endoscopic holmium laser lithotripsy (PTEHL).

Characteristics	Number (%) or mean ± SD
Pre-PTEHL drainage	
Yes	24 (48)
No	26 (52)
Mean duration from drainage to PTEHL (days)	10.5 ± 6.3
Sheath size	
16F	42 (84)
18F	8 (16)
Number of PTEHL sessions	
Single session	48 (96)
Two sessions	2 (4)
Technical success rate	
After first session	48 (96)
After second session	50 (100)
Stone clearance status	
Complete clearance	36 (72)
Near-complete clearance	14 (28)
Residual stones	0 (0)
Mean hospital stay (days)	14.8 ± 7.97

PTEHL: Percutaneous transhepatic endoscopic holmium laser lithotripsy; Complete clearance: refers to no visible residual stones on final cholangiography.

**Table 3:** Factors associated with complete stone clearance.

Variable	Univariable analysis		Multivariable analysis	
	OR (95% CI)	P value	OR (95% CI)	P value
Pre PTEHL drainage				
Yes	1			
No	0.75 (0.22–2.60)	0.65		
Cholangitis				
No	1		1	
Yes	6.0 (1.17–30.73)	0.003	6.57 (1–43.27)	0.05
Biliary stricture				
No	1		1	
Yes	4.67 (1.25–17.44)	0.022	3.05 (0.51–18.27)	0.222
Number stone				
Single	1			
Multiple stones	4.80 (0.94–24.62)	0.06		
Stone location				
CBD only	1		1	
CBD+IHBD or IBHD	23.0 (2.69–196.40)	0.004	12.65 (1.21–125.00)	0.034
Stone size				
< 2 cm	1		1	
≥ 2 cm	4.67 (1.25–12.40)	0.022	3.22 (0.56–18.42)	0.189

OR: odds ratio; CI: confidence interval; CBD: common bile duct; IHBD: intrahepatic bile duct; PTEHL: Percutaneous transhepatic endoscopic holmium laser lithotripsy.

Adverse events: we recorded 13 patients (26%) with post-PTEHL adverse events. Among them, 1 patient had a biliopleural fistula requiring drainage. Mild hemobilia occurred in 8 patients (16%) and resolved spontaneously after 2–3 days of observation. Additionally, 2 patients (4%) had mild pleural effusion, 1 patient (2%) developed a perihepatic fluid collection, and 1 patient (2%) experienced unexplained post-procedural vomiting. According to the CIRSE classification, 1 patient (2%) experienced a moderate complication (Grade 3), while 12 patients (24%) experienced minor complications, including 5 patients with Grade 2 (10%) and 7 with Grade 1 (14%).

The average hospital stay was 14.8 ± 8.0 days (range: 3–37 days), with no mortality. Patients with cholangitis had a longer hospital stay (17.6 ± 8.0 days) compared to those without cholangitis (10.6 ± 5.7 days), with the difference being statistically significant (p = 0.001), (Table 4). Drainage catheters were successfully removed in all patients, with no cases of readmission due to biliary-related symptoms during the 3-month follow-up period.

**Table 4:** Procedure-related complications and CIRSE classification.

Complication	Number of Patients (%)
Type of complication	
Hemobilia	8 (16)
Pleural effusion	3 (6)
Perihepatic fluid collection	1 (2)
Severe post-procedural vomiting	1 (2)
CIRSE classification	
Minor	12 (24)
1	7 (14)
2	5 (10)
Moderate	1 (2)
Major	0 (0)

## Discussion

The increasing incidence and severity of biliary stone disease in the elderly present substantial challenges in clinical decision-making. In Lee’s study, the mortality rate of acute cholangitis due to choledocholithiasis was 4.5%, while Nassar reported a rate as high as 18.87% [6,16]. These figures highlight the need to prioritize procedural safety in this population. In our study, PTEHL achieved a 96% single-session success rate and 100% overall technical success. Complete stone clearance was obtained in 72% of patients, and no surgical conversion or alternative treatment was required. Most complications were mild and self-limiting, with only one major event (2%) a biliopleural fistula requiring drainage and no mortality occurred.

Surgical management remains complex in elderly patients [8]. Ho identified coagulopathy (OR: 3.74), electrolyte imbalance (OR: 2.89), and liver disease (OR: 1.89) as predictors of higher postoperative mortality [1]. Watt et al, in a meta-analysis, reported an overall postoperative complication rate of 25.17% (95% CI: 18.03–33.98%) in the elderly [2].

ERCP is generally the first-line treatment, particularly for CBD stones. However, in Zang et al’s study of 156 patients aged ≥85 years (stone size 16.8 ± 4.8 mm), ERCP failed in 23.7% of cases [4]. Risk factors for failure included stones >10 mm, multiple stones, APACHE II and ASA scores ≥3, and emergency ERCP [4]. Cagir et al compared ERCP complication rates between younger and elderly patients (≥75 years), reporting similar rates (9.8% vs. 7.1%, p = 0.292), though elderly patients had longer hospital stays and worse outcomes [17]. Postoperative or age-related anatomical changes further complicate ERCP access and success [17]. Laparoscopic CBD exploration is a feasible alternative when ERCP fails. In Jia’s study, early laparoscopic intervention (<8 hours) in patients ≥65 years with cholangitis yielded a complication rate of 4.08%, favoring timely surgery [18]. However, a meta-analysis by Delgado covering 12 studies (3,791 patients, including 1,411 elderly) showed significantly higher mortality (OR: 3.42, P = 0.04), complications (OR: 1.60, P = 0.01, I^2^ = 52%), and pneumonia (OR: 4.37, P < 0.01) in elderly surgical patients. Prior surgeries and recurrent stones further complicate operative management [19].

Percutaneous biliary endoscopy (PBE) is increasingly adopted. Guan et al reported technical success rates of 98.3% for CBD and 100% for gallbladder endoscopy using PBE [12]. In a multicenter study by Alec Jost et al, electrohydraulic lithotripsy (EHL) and laser lithotripsy achieved technical success rates of 92.4% and 98.4%, respectively, though residual stones remained in 44.0% and 55.1% of cases after the first session [13]. Kim et al used EHL or YAG laser through a 12F sheath in high-risk acute cholecystitis patients, achieving 100% technical and 95.2% clinical success [20]. Facciorusso et al. analyzed 19 randomized controlled trials including 2,752 patients with large bile duct stones (>10 mm) and found that cholangioscopy was the most effective treatment modality compared with alternative approaches, especially for stones >15 mm [21]. In comparison, our study achieved 100% technical success, fourteen patients (28%) had small residual peripheral stones located deep within the terminal branches of the peripheral subsegmental bile ducts. These stones were asymptomatic and situated in locations inaccessible to the cholangioscope or interventional devices. Such sites would also be difficult to access using other treatment modalities, including surgery or ERCP. No patients required repeat PTEHL or alternative intervention due to stone migration into larger bile duct branches or the common bile duct.

In the multivariable analysis, cholangitis (*p* = 0.05) and the presence of both intrahepatic and extrahepatic stones (*p* = 0.034) remained statistically significant predictors of complete stone clearance. However, for stone location, the 95% confidence interval of the odds ratio was very wide (OR = 12.65; 95% CI: 1.21–125.00), suggesting an imprecise and potentially unstable estimate. This may be attributable to the small number of patients or outcome events in certain stone-location subgroups. Therefore, this finding should be interpreted with caution and requires confirmation in larger studies

The overall complication rate was low in this cohort. One of 50 patients (2%) experienced a moderate complication (CIRSE Grade 3), while minor events were managed conservatively without the need for additional intervention. In Lamanna’s study, complications occurred in 33% of patients (4/12), including one case of cholangitis [22]. Jost et al reported moderate to severe complications in 3.3% − 4.3%. PTEHL appears to offer a safer profile in elderly patients, avoiding general anesthesia and reducing the risk of major postoperative events [13].

Our study has several limitations. First, the relatively small sample size (n = 50) limits the generalizability and statistical power of the findings. Furthermore, the inclusion of multiple predictors in the multivariable model raises concerns regarding overfitting, which may compromise the stability and robustness of the estimated associations.. Second, there was no control group or comparative arm such as ERCP or laparoscopic stone removal, which would have allowed for a more comprehensive evaluation of safety and efficacy. Third, the study lacked long term follow-up data to assess stone recurrence or late complications. Lastly, the procedures were conducted at a specialized center with extensive experience in PTEHL and biliary interventions, which may limit external applicability.

## Conclusion

PTEHL may represent a feasible minimally invasive treatment option for elderly patients with biliary stones; however, comparative studies are needed to determine its relative safety and effectiveness versus other treatment modalities.
